# Pancytopenia Resulting From Low-Dose Methotrexate Use: A Diagnostic Challenge

**DOI:** 10.7759/cureus.15193

**Published:** 2021-05-23

**Authors:** Syed Wajih Ul Hassan

**Affiliations:** 1 Medical Intensive Care Unit, Darul Sehat Hospital, Karachi, PAK

**Keywords:** reticulocyte count, rheumatoid arthritis, felty’s syndrome, bone marrow suppression, nsaid, hemophagocytic lymphohistiocytosis (hlh), bone marrow biopsy, methotrexate-induced pancytopenia

## Abstract

Rheumatoid arthritis (RA) is a common autoimmune disease primarily affecting small joints which leads to crippling erosion of the articular cartilage and bone. It is associated with complications related to both its disease course and treatment. Methotrexate (MTX) is a folate antagonist responsible for modulating cell-specific signaling pathways and inhibiting the proinflammatory properties of major cell lineages involved in the pathogenesis of RA. It is considered to be the first-line agent in RA because of its disease-modifying ability and safety profile at low doses. This case report discusses how a middle-aged female presented with severe bone marrow suppression secondary to MTX toxicity, an unusual presentation at the usual low-dose regimen. Her presentation overlapped with several other conditions, especially with Felty’s syndrome, a rare complication of RA, characterized by the triad of splenomegaly, neutropenia, and RA. Other differentials included hemophagocytic lymphohistiocytosis, hematologic neoplasms, drug reaction, and infection. Therefore, it was essential to exclude all possible differentials before initiating therapy. We found the corrected reticulocyte count coupled with a good response to leucovorin to be an effective way to differentiate MTX-induced pancytopenia from other possible hematologic diagnoses without the use of a bone marrow biopsy. Additionally, our case incidentally demonstrated a potential interaction between piperacillin/tazobactam and MTX.

## Introduction

Rheumatoid arthritis (RA) is an autoimmune disease causing inflammatory polyarthritis primarily of the small joints leading to crippling erosion of the articular cartilage and bone. Common complications include joint deformity and immobility; however, extraarticular manifestations are also possible. An uncommon extraarticular complication of this disease is Felty’s syndrome (FS), which is characterized by the triad of neutropenia, splenomegaly, and RA. It occurs in less than 1% of the people with RA [1]. However, the extraarticular manifestations of RA are generally associated with higher mortality [2]. Therefore, accurate recognition and treatment of the disease are vital to patient survival.

Methotrexate (MTX) is a folate antagonist responsible for modulating cell-specific signaling pathways and inhibiting the proinflammatory properties of major cell lineages involved in the pathogenesis of RA. It is considered to be the first-line agent in RA because of its disease-modifying ability and safety profile at low doses. However, at higher doses, it is known to cause adverse effects such as pancytopenia, liver toxicity, and renal failure [3].

Our patient was a middle-aged female on low-dose MTX therapy for RA, who presented with pancytopenia, an unusual presentation at the standard low-dose regimen. The presence of neutropenia and splenomegaly complicated the diagnosis for this case as FS had become a possibility. The fever and pancytopenia also begged the exclusion of hematologic malignancies and sepsis as possible causes.

A common practice is to use a bone marrow biopsy to identify the cause of low cell counts and the enlarged spleen. Unfortunately, this practice can be cumbersome and cause the patient undue pain.

I hope to discuss the use of “corrected reticulocyte count” and the initiation of filgrastim and leucovorin rescue therapy as an initial effort that can potentially defer the use of bone marrow biopsy in cases presenting with fever and pancytopenia, such as ours. In addition, I aim to briefly discuss the incidental findings of this case and its implications.

## Case presentation

A 45-year-old female presented to the emergency department with complaints of fever, diarrhea, vomiting, and anuria. She was a known case of seropositive RA since 2008. Her list of regular medications comprised injection MTX 15 mg weekly, oral leflunomide (LFE) 20 mg daily, oral prednisolone 5 mg daily, oral folic acid 5 mg daily, oral pantoprazole 40 mg daily, and vitamin D supplementation. She occasionally used non-steroidal anti-inflammatory drugs (NSAIDs) to control any additional joint pains.

On examination, she had a temperature of 102°F but was otherwise vitally stable and oriented. She had subconjunctival pallor, yellowish sclera, stomatitis, and a 7 × 7 cm carbuncle on her left scapular region. Abdominal examination revealed a tender liver that was palpable 2 cm below the costal margin, but the spleen could not be palpated. Musculoskeletal examination revealed stiffness in bilateral wrists and metacarpophalangeal and interphalangeal joints. Per vaginal examination revealed multiple superficial ulcers on the vulva.

Her initial blood workup showed hemoglobin (Hb) of 7.7 g/dL, mean corpuscular volume of 75 fl, total leucocyte count (TLC) of 2,100/µL (absolute neutrophil count: 1,680/µL), platelets of 133,000/µL, hematocrit of 25%, and corrected reticulocyte count of <2%. Her renal functions demonstrated urea 60 mg/dL and creatinine 3.5 mg/dL. Baseline creatinine was 0.7 mg/dL. Her liver function tests showed total bilirubin at 4.8 mg/dL, direct bilirubin 2.8 mg/dL, indirect bilirubin 2.0 mg/dL, serum glutamic pyruvic transaminase (SGPT) 11 U/L, alkaline phosphatase (ALP) 477 U/L, and gamma-glutamyl transferase (GGT) 70 U/L. Her viral markers for hepatitis A, B, C, and E were non-reactive. Additional blood tests revealed HbA1c of 10.4%, lactate dehydrogenase (LDH) 475 U/L, ferritin 1,398 µg/L, vitamin B12 354.9 pg/mL, red blood cell folate 4.92 ng/mL, lactic acid 11.0 mg/dL, and 24-hour urinary protein 90 mg. Direct Coombs test and antinuclear antibody (ANA) were positive. Peripheral smear revealed normochromic, anisocytosis, dimorphic picture with no abnormal cells. Her electrocardiogram (ECG), echocardiography (ECHO), and chest X-ray were all unremarkable. Urinalysis was positive for glucose, epithelial cells, and yeast cells; however, bile pigments were negative and urobilinogen levels were normal. Urine culture and sensitivity showed growth of *Candida* species, but blood cultures showed no growth after 48 hours of incubation. Ultrasound showed a liver measuring 21.8 cm with an altered parenchymal echo pattern and a bulky spleen measuring 13.7 cm.

Given the patient history, physical examination, and investigations, a provisional diagnosis of MTX-induced pancytopenia secondary to NSAID-induced acute kidney injury (AKI) was made. MTX and LFE were immediately stopped, and she was started on injection leucovorin 15 mg thrice daily. Given the presence of fever, carbuncle, and yeast cells on urinalysis, she was provided broad-spectrum antibiotic and antifungal cover as per the hospital protocol, which included injection piperacillin/tazobactam 4.5 g thrice daily, injection imipenem 250 mg thrice daily, and oral fluconazole 50 mg once daily. She was also transfused two red blood pack cells and started on intravenous (IV) hydration.

On day two, TLC reduced to 900/µL, and injection filgrastim 300 mg subcutaneously once daily was started. Piperacillin/tazobactam was switched to injection clindamycin 600 mg thrice daily as per expert consultant opinion. Following two days of IV hydration, she began to produce approximately 1,600 mL of urine per day. Her cell counts improved by day five (Hb: 9.4 g/dL, TLC: 4,600/µL, absolute neutrophil count: 3,772/µL, and platelets: 167,000/µL) of therapy and filgrastim was stopped. As cell counts improved following therapy, bone marrow biopsy was deferred. Her carbuncle was excised on day 10 of her hospital stay. On day 13, her labs showed urea 32 mg/dL, creatinine 2.0 mg/dL, total bilirubin 1.1 mg/dL, direct bilirubin 0.6 mg/dL, indirect bilirubin 0.5 mg/dL, SGPT 11 U/L, ALP 552 U/L, and GGT 45 U/L.

## Discussion

The presence of fever and splenomegaly presented a diagnostic challenge in our patient. The fever might have been a result of RA, an infection, drug reaction, hemophagocytic lymphohistiocytosis (HLH), or hematologic neoplasms. These differential diagnoses had to be carefully considered and logically excluded given their high mortality and morbidity.

Our patient was provisionally diagnosed as MTX-induced pancytopenia secondary to AKI given the absence of splenomegaly on clinical examination and the presence of risk factors such as polypharmacy and irregular follow-up. The incidental finding of splenomegaly on ultrasound, however, cast the diagnosis in doubt. The diagnostic triad for FS includes RA, splenomegaly, and neutropenia, which were all present in our patient. The presence of a high LDH, elevated indirect bilirubin, ANA, and a positive direct Coombs test gave an initial impression of hemolytic anemia which was in line with FS. However, a low corrected reticulocyte count clarified that the low Hb was due to underproduction instead of increased lysis. A peripheral smear with no schistocytes, spherocytes, or any other abnormal cells supported these findings. A decrease in red cell production was in line with MTX-induced pancytopenia instead of FS and allowed us to safely discontinue MTX, thereby avoiding any unwarranted therapies with biological disease-modifying antirheumatic drug (DMARD).

The presence of fever, splenomegaly, pancytopenia, and high serum ferritin also alerted us to the possibility of HLH and a possible hematologic neoplasm. The HLH-2004 criteria include either a genetic diagnosis or five out of eight clinical and lab parameters: fever, splenomegaly, bicytopenia, hyperferritinemia, hypertriglyceridemia, hypofibrogenimea, and sIL2r of >2,400 U/mL. However, our patient only met four out of eight of the HLH-2004 diagnostic criteria, and the decreasing C-reactive protein (CRP) essentially excluded the diagnosis. The possibility of lymphoma or leukemia was excluded based on the absence of weight loss, lymphadenopathy, low lymphocyte counts, and lack of abnormal cells on the peripheral smear.

The diagnosis of MTX-induced pancytopenia was proven by the effective response to leucovorin and filgrastim therapy and withholding MTX. Improving cell counts and decreasing CRP were used as trends to monitor the effectiveness of therapy. Although a bone marrow biopsy is an important step in the diagnostic pathway to exclude myeloid or lymphoid proliferation in patients with pancytopenia, it can be a painful procedure with potentially unnecessary complications in a low-risk individual such as our patient [4]. The bone marrow biopsy was deferred given the positive response to therapy.

MTX is considered the first-line DMARD for treating RA. When given at the standard 7.5-25 mg weekly dose, adverse drug reactions are rare [5]. However, hypoalbuminemia, renal failure, and polypharmacy were risk factors for developing MTX toxicity, which were present in our patient [6]. MTX toxicity occurs as both an idiosyncratic reaction and dose-dependent reaction. Given the chronic history and risk factors of our patient, this reaction most likely occurred due to cumulative doses of the drug. The MTX drug level was not obtained as the literature shows adverse effects are a function of the duration of suprathreshold levels instead of peak levels achieved [7]. Our case demonstrated the findings of fever, vomiting, diarrhea, stomatitis, mucocutaneous ulcers, and pancytopenia, which are classical findings of MTX-induced toxicity [8].

Combination therapy of MTX and LFE has been propagated for recalcitrant disease [9]. Given the possibility of pharmacodynamic synergism, one would assume the combination therapy to have worse side effects than monotherapy. However, studies showed otherwise, with the combination only eliciting mild side effects such as gastrointestinal upset and liver enzyme elevation [10]. A few case reports demonstrated the presence of pancytopenia like ours [11]. No case reports were found demonstrating renal toxicity of low-dose MTX or LFE.

MTX-induced renal injury results from using MTX at high doses as in the treatment of malignant neoplasms. AKI secondary to low-dose MTX is uncommon and usually the result of concomitant use of NSAIDs [12]. NSAIDs reduce blood flow to the kidney and make them vulnerable to a pre-renal insult such as hypovolemia resulting from diarrhea. Initially, it seemed like this might have been the case in our patient. Given that her blood pressure never acutely dropped, and serum lactic acid levels were normal, a pre-renal injury seemed unlikely. A more plausible explanation might be interstitial nephritis given her use of NSAID, amoxicillin, and pantoprazole [13]. Other supportive findings included the presence of sub-nephrotic proteinuria and improvement of renal function after drug withdrawal over two weeks.

There are no formal guidelines on how to treat MTX-induced pancytopenia. However, according to a literature review, leucovorin plays a significant role in reversing MTX-induced myelosuppression [14]. Likewise, a review found filgrastim to improve cell counts in febrile neutropenia [15]. The average time to recovery of cell counts was five days in a case report [16]; however, a duration of four days was noted in this study. This might be explained by the fact that our patient had sufficient folate levels on presentation and was initiated immediately on leucovorin rescue after clinical suspicion. I also recognize that the use of piperacillin/tazobactam as an initial empiric antibiotic might have had a negative impact on our therapy, as cell counts drastically dropped following a single dose. Only a handful of case reports have highlighted this potential drug-drug interaction between high-dose MTX and piperacillin/tazobactam [17], none with low-dose MTX.

In this case, an unusual finding was the presence of a cholestatic pattern of liver function tests, with a normal SGPT. MTX has been implicated in causing elevation of aminotransferases, portal fibrosis, and cirrhosis. The presence of jaundice, elevated ALP, and GGT along with splenomegaly was suggestive of liver fibrosis; however, an ultrasound revealed a normal common bile duct, intrahepatic duct, and portal vein diameter. Furthermore, risk factors for the development of liver fibrosis include diabetes and obesity, both of which were present in our patient. The patient should ideally have been evaluated with ultrasound elastography for further classification; however, this was not available in our facility. Bilirubin levels and GGT showed a gradual improvement along the course of the patient’s stay.

## Conclusions

Fever and pancytopenia secondary to MTX can present a diagnostic challenge in patients with splenomegaly who are known cases of RA. However, corrected reticulocyte count is a simple yet effective test in differentiating FS from bone marrow suppression secondary to MTX. An adequate response to filgrastim and leucovorin and a low lymphocyte count on complete blood count essentially ruled out other remaining hematologic diagnoses and the need for bone marrow biopsy. It is worth noting that piperacillin/tazobactam has potentially serious drug-drug interactions with MTX and should be avoided in patients already on MTX. Concomitant use of NSAIDs should also be avoided as much as possible in MTX users given the potential for AKI and secondary bone marrow suppression even at low doses of MTX.
